# Cleaving Method for Molecular Crystals and Its Application to Calculation of the Surface Free Energy of Crystalline β-d-Mannitol at Room Temperature

**DOI:** 10.1021/acs.jpca.2c00604

**Published:** 2022-03-24

**Authors:** Nicodemo Di Pasquale, Ruslan L. Davidchack

**Affiliations:** School of Mathematics and Actuarial Science, University of Leicester, University Road, Leicester LE1 7RH, United Kingdom

## Abstract

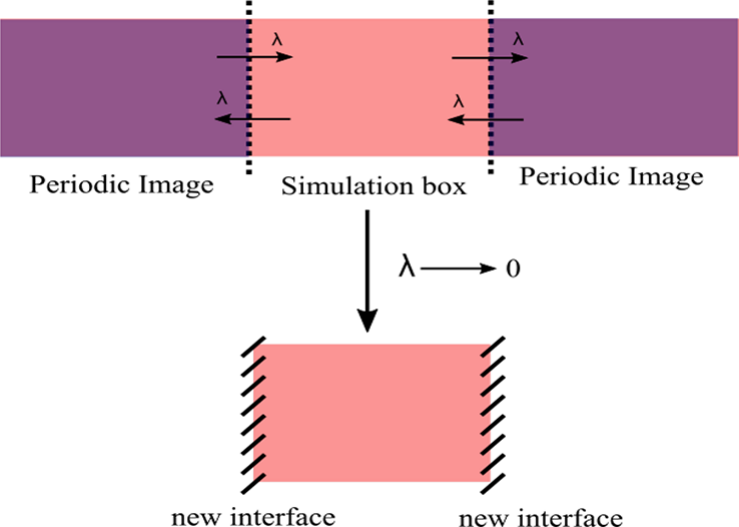

Calculation of the surface free energy (SFE) is an important application of the thermodynamic integration (TI) methodology, which was mainly employed for atomic crystals (such as Lennard–Jones or metals). In this work, we present the calculation of the SFE of a molecular crystal using the cleaving technique which we implemented in the LAMMPS molecular dynamics package. We apply this methodology to a crystal of β-d-mannitol at room temperature and report the results for two types of force fields belonging to the GROMOS family: all atoms and united atoms. The results show strong dependence on the type of force field used, highlighting the need for the development of better force fields to model the surface properties of molecular crystals. In particular, we observe that the united-atoms force field, despite its higher degree of coarse graining compared to the all-atoms force field, produces SFE results in better agreement with the experimental results from inverse gas chromatography measurements.

## Introduction

Determination of the surface properties of materials is a problem probably as old as the development of thermodynamics itself, which was formalized by Gibbs^1^ for solid and liquid interfaces. Interest in the modeling of interfaces and determination of their properties stems from the fact that many physical phenomena (freezing, nucleation, confinement) and technological processes (casting, welding, formulation) involving solid phases require detailed knowledge of the structure and thermodynamic properties of interfaces between the solid and other phases. As an example, in pharmaceutics production, the behavior of formulations with respect to binder–drug adhesion,^2^ powder flow, and compaction^3^ can be related to knowledge of the surface properties.^4^

Even though the importance of surface properties is well known, exact determination of these properties in experiments is still very difficult due to the strict control necessary on the experimental parameters or the difficulty to estimate some characteristics which can affect the measurements, such as the irregularity or porosity of the surfaces. On the other hand, in silico experiments with molecular dynamics (MD) allow one to calculate these quantities from their thermodynamic definitions, giving access to measurements and underlying mechanisms of surface characterization.

One of the key thermodynamic properties of a surface is its surface free energy (SFE). Among the various methods to calculate the SFE in molecular simulations, the *cleaving method* provides direct and accurate results. The cleaving methodology for solid–liquid interfaces proposed in the 1980s by Broughton and Gilmer^5^ and later refined in refs (6) and (7) belongs to the wider class of thermodynamic integration (TI) methods.^8^ In TI, a continuous thermodynamic path is defined between two points in the space of thermodynamic parameters of the system. The free energy difference between these points is determined by the reversible work needed to transform the system from the initial point to the final point and is calculated by integrating the ensemble average of some configuration-dependent function (e.g., potential energy) with respect to the switching parameter that parametrizes the path. The path independence of the reversible work ensures that any path connecting the two points can be chosen, even including nonphysical intermediate systems.^9,10^

Until recently, calculation of the SFE using TI was mainly limited to metals,^11^ simple atomistic solids (e.g., Lennard–Jones crystal),^6,7,12−14^ and water.^15,16^ Extension of TI techniques to molecular crystals includes calculation of the free energy of molecular crystals by defining a thermodynamic path between simple Einstein crystals and the system under study.^17,18^ In this work, we present an extension of the cleaving methodology to calculation of the SFE of organic molecular crystals, such as mannitol. We determine the SFE of different surfaces of the mannitol crystal at room temperature. We consider different force fields and experimental crystal structures in order to investigate their effect on the value of the mannitol crystal SFE.

We study the crystalline form of d-mannitol (C_6_H_14_O_6_), a hexahydric sugar alcohol, which has three polymorphic forms.^19^ In this work, we focus on the most thermodynamically stable orthorhombic form, β-d-mannitol. d-Mannitol is a common pharmaceutical excipient, included in a variety of formulations, such as chewable tablets, powder granules, and moisture-sensitive active pharmaceutical ingredients (APIs), thanks to its nonhygroscopicity, noncariogenicity, and cooling property in the oral cavity.^20^

Due to its importance as a pharmaceutical excipient, mannitol has been widely studied, and a number of experimental determinations of the surface energy are reported in the literature. In particular, the surface energy of mannitol was measured using inverse gas chromatography (IGC).^4^ In IGC, a crystalline sample of the studied material is packed into a column and its surface properties are analyzed by measuring the retention time of known vapor probes which are injected at infinite dilution. By varying the compound in the vapor phase, it is possible to determine the SFE from the difference of interaction of the crystal surface with different compounds. In particular, the “dispersive component” of the SFE is determined from the measurements with a series of linear *n*-alkane probes of increasing length (heptane, octane, nonane, decane), while the “acid-based” or “polar” component is obtained with probes like toluene, acetone, ethanol, etc.^21^

The experimental results obtained through IGC provide a reference for the computational results we present here. Besides, MD calculations allow one to determine the contribution to the SFE of the Lennard–Jones and Coulomb components of the force field, and it is interesting to compare these contributions with, respectively, the dispersive and polar components of the surface energy measured by IGC.

The paper is organized as follows. We start by discussing a modification of the cleaving method for molecular crystals, deriving all of the relevant quantities needed in the calculation of the SFE. We then give the details of the MD calculations and present the results followed by a comparison with the experimental results reported in Ho et al.^4^ Finally, we conclude with comments about the relative merits of the all-atoms and united-atoms force fields for mannitol and their effectiveness in predicting the surface thermodynamics of the mannitol crystal.

## Thermodynamic Integration

The SFE in this work is determined using a simplified version of the cleaving method described in detail in refs (6) and (7). Here, we will give a brief outline. The simplification is related to the fact that we are calculating the free energy of a solid surface (i.e., a solid–vacuum interface), so the steps related to the cleaving of the liquid phase are not needed.

Moreover, we take advantage of the fact that at the temperatures we are considering the molecules do not leave the crystal lattice. This, in turn, translates into not needing an external potential to prevent the molecules from crossing the cleaving plane. Therefore, of the original four steps of the cleaving method,^6,7^ we need only a single step: the creation of crystal surfaces by turning off the interactions among atoms across the cleaving plane. However, the simplified path we just described does not reduce to a simple translation of the known cleaving methodology to this particular system. Application of the cleaving methodology to the molecular crystals that we are presenting here include analysis and adaptation of the original technique that we are going to detail in this section as well as modifications to the algorithm needed to calculate the scaled and unscaled interactions (see SI for more details). In the future, we can build on this generalization of the cleaving technique in order to extend this approach to molecular crystals in contact with liquids.

**Figure 1:**
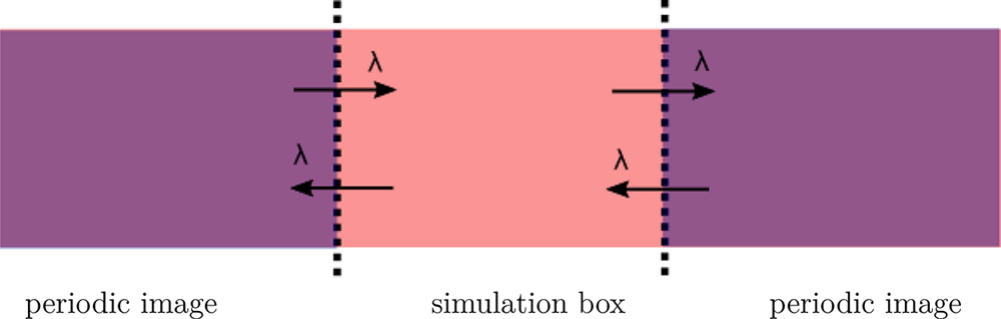
Sketch of the initial setup for the cleaving method applied to the crystal surface. Arrows represent the scaled interactions which are multiplied by the coupling parameter λ. Dotted lines represent the new surfaces. When λ = 0, the simulation box is not interacting with the periodic images anymore.

The setup for this system is shown in Figure 1. Let us consider a system in two different thermodynamic states: the bulk, i.e., an infinite crystal without surfaces, and the slab, a crystal with two parallel surfaces. In MD simulations, the bulk is modeled in a simulation box with periodic boundary conditions (PBC) in the three directions,^22^ while the slab is modeled in a box with PBC only in the two directions tangent to the surfaces. It is important to highlight here that, in practice, the simulation of the slab is carried out with the PBC in all directions. However, in the slab configuration, the system does not interact with its periodic images in the direction normal to the surfaces, that is, the system behaves effectively as having PBC in only two directions. In the rest of the work we will refer to the slab configuration as the configuration with no PBC in one direction. However, this description must be understood in the sense just defined (i.e., as removing interactions between the system and its periodic images in one direction), which let us construct a continuous thermodynamic path between the bulk and the slab configurations.

Both systems have the same number of molecules, and the thickness of the slab system is sufficiently large so that the region inside the slab sufficiently far from the surfaces has properties identical (within statistical uncertainty) to those of the bulk system.

The bulk and slab configurations represent the end points of a continuous thermodynamic path which is parametrized by λ ∈ [0, 1] with λ = 1 representing the bulk and λ = 0 representing the slab.

Starting with the bulk system, the surfaces in the slab system are created by “switching off” the intermolecular interactions on the two sides of a plane placed between two adjacent layers of the crystal with a specified crystallographic orientation. For this reason, the system potential energy, , is also a function of λ, that is, , where **r** represents the configurational state of the system. As such, the intermolecular contribution to  is evaluated in the bulk configuration, *U*_*b*_(**r**), whereas the intermolecular contribution to  is evaluated in the slab configuration, *U*_*s*_(**r**). Note that the majority of intermolecular force fields consist of Lennard–Jones (LJ) interactions between atomic nuclei (or atomic groups centroids) and Coulomb (C) interactions between residual charge sites. Therefore, we can write explicitly
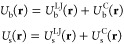
1To this we must add the intramolecular interactions, *U*_i_(**r**), which represent the interactions among atoms within the same molecule, including bond, angle, dihedral, etc., that determine the structure and rigidity of the molecule. Since we are assuming that molecules do not break up during creation of the surface, the function *U*_i_(**r**) does not include any λ dependence.

The total potential energy  can be therefore written as
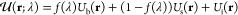
2where we included the scaled interactions terms *U*_b_(**r**) and *U*_s_(**r**) and the nonscaled term describing the intramolecular interactions *U*_i_(**r**). The continuous function *f*(λ) must be such that *f*(0) = 0 and *f*(1) = 1. We choose *f*(λ) as
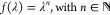
3In this work, we used *n* = 4; however, we also tested *n* = 2 and 3 for consistency, obtaining consistent values of the SFE for all of the cases considered. A relatively high value of *n* is necessary to reduce the so-called “LJ catastrophe”,^8^ which happens when λ is close to zero. In this situation, the repulsive forces between atoms at small distances are scaled by a near-zero value of λ, thus allowing atoms to get very close, where the very steep gradient of the LJ potential significantly magnifies the small errors of the numerical integrator applied to the equations of motion. As a result, huge repulsive forces may be experienced by atoms that approach too close, causing instability of the MD simulations.

In addition to using a high value of *n*, we also address this problem by replacing the LJ potential at very short distances, *r* < *r*_*m*_, by a polynomial *p*(*r*), such that the transition from LJ to *p*(*r*) at *r* = *r*_*m*_ is smooth. The modified LJ potential reads
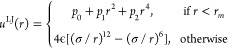
4where *r* is the distance between interacting LJ sites and the polynomial coefficients *p*_0_, *p*_1_, and *p*_2_ are determined by imposing the two times differentiability at *r* = *r*_*m*_. If we choose *r*_*m*_ to be sufficiently small (such that the distance between interacting LJ sites is never smaller than *r*_*m*_ when λ = 1) then the initial and final states of the system are unaffected by this modification of the LJ potential and neither is the reversible work calculated along the TI path between these states. In this work, we used *r*_*m*_ = 0.5σ, which is sufficiently small compared to the minimal distance between LJ sites during the simulation with λ = 1.

The SFE γ is then obtained by integration^8^
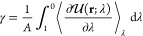
5where *A* is the area of the created surfaces and ⟨···⟩_λ_ represent the ensemble average, which depends on λ through the total potential in eq 2. When the SFE is calculated in this way, it represents the work per unit area needed to create a new surface by separating molecules in the bulk configuration at the cleaving plane.

Note that, in general, the two surfaces created in the cleaving process might have different structures, so their SFEs might also be different. In this case, the γ calculated in eq 5 represents the average SFE of the two surfaces. However, because of the symmetry of the mannitol molecule, the two created surfaces are structurally identical for all of the surface orientations considered in this work.

We want to highlight here that we are not making any assumptions on the type of interactions, i.e.,  can contain the pairwise, three-body, four-body, embedded atom, etc., interactions between sites collectively denoted as **r**. In MD simulations of polymers and biomolecules the highest order considered is the four-body potential, describing dihedral angles in the molecules. The generalization of the cleaving method to these types of interactions is one of the novelties presented here.

Using eqs 2 and 3 we can write the integrand in eq 5 in the form
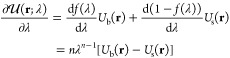
6The split of the intermolecular interactions into the LJ and Coulomb parts, as in eq 1, allows us to evaluate separately the contribution of these interactions to the value of the SFE γ = γ^LJ^ + γ^C^ where
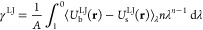
7a
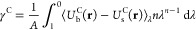
7bSince the LJ interactions are associated mainly with the van der Waals forces while the Coulomb component is linked with the hydrogen bonds formed between molecules, it is interesting to compare this split with that between the dispersive and polar contributions to the SFE obtained from the IGC measurements^23^
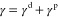
8While elucidating the link between the LJ and the Coulomb contributions to the calculated SFE and the IGC measurements of dispersive and polar components will require further investigation, in this work we report a comparison between the components of the SFE and the reported experimental results from ICG. It is important to note, however, that due to the presence of other (intramolecular) interactions, the LJ and Coulomb contributions to the SFE cannot be fully decoupled, that is, equilibration of the system at the intermediate λ values will lead to deformation of the molecules near the surfaces of the slab compared to those in the bulk. Such deformations will increase with decreasing λ and influence both the LJ and the Coulomb integrands in eqs 7a and 7b. Nevertheless, because mannitol molecules are relatively stiff, their deformation is not very large, so the coupling between the LJ and the Coulomb contributions is not expected to be very large.

## Computational Details

The simulated system is the orthorhombic β-crystal of d-mannitol with five different surface orientations: (100), (010), (001), (011), and (120). The surfaces (010), (011), and (120) are observed experimentally and thus are expected to have lower SFE compared to (100) and (001). The system was built by replicating the unit cell 4 times in the *x* direction, 2 times in the *y* direction, and 7 times in the *z* direction.

The simulations were performed using the LAMMPS simulation package^24^ with two versions of the GROMOS force field.^25,26^ In the first version, labeled “AA”, the molecules are modeled in the all-atoms (including hydrogens) configuration with charges modified using the ATB package.^27,28^ In the second version, labeled “UA”, molecules are modeled using the united-atoms description (i.e., the carbon–hydrogen groups are represented by a single interaction site) with the charges optimized specifically for the mannitol.^29^ We used a time step of 0.5 fs for the AA model and 1 fs for the UA model. The LJ cutoff is 12 Å, and we mixed the LJ interactions using the geometric rules. We calculated the Coulombic interactions via the Wolf summation method^30^ as presented in ref (31) with 14 Å cutoff and the damping parameter α = 0.075.^32^ The setup described here was tested against a calculation with Ewald summation (with a tolerance on the force of 10^–5^), obtaining consistent results between the two methodologies.

For the structure, we downloaded from the Cambridge Structural Database (CSD)^33^ three different versions of the mannitol unit cell, identified by the acronyms DMANTL09,^19^ DMANTL07,^34^ and DMANTL,^35^ each with the same crystallographic space group *P*2_1_2_1_2_1_. Each version of the unit cell was replicated in three directions to create a crystal sample of approximate size 35 Å × 35 Å × 40 Å containing 224 molecules. The samples were equilibrated at 300 K and zero pressure in both AA and UA GROMOS force fields for 3 ns using a Nosé–Hoover thermostat and an anisotropic barostat. We found that some of the systems developed various types of crystal defects during the equilibration, so these structures could no longer be viewed as the β-crystal. Of the three variants, only DMANTL09 equilibrated without defects with the AA force field and only DMANTL equilibrated without defects with the UA force field. For this reason, we will consider in this work the AA force field only with the DMANTL09 structure and analogously for the pair UA and DMANTL. The average unit cell parameters for the two selected structures measured during the last 1 ns of the equilibration run and a comparison with the experimental values of the lattice constants and densities are reported in Table 1.

**Table 1:** Lattice Parameters and Densities for the Three Structures of Mannitol Considered in This Work^a^

	DMANTL lattice parameter (Å)	
	experimental^35^	UA
*a*	8.672	9.05(4) [4%]
*b*	16.875	17.78(7) [6%]
*c*	5.560	5.839(1) [5%]
density (kg/m^3^)	1487	1288(5) [−13%]

	DMANTL09 lattice parameter (Å)	
	experimental^19^	AA
*a*	8.580	8.33(2) [−3%]
*b*	16.795	16.562(1) [−1%]
*c*	5.538	5.490(6) [−1%]
density (kg/m^3^)	1484	1572(5) [6%]

a The experimental values are taken from the cited literature. The simulation values are from the equilibrated crystal structures using the UA and AA force fields. The numbers in parentheses represent the statistical error in the last digit shown, whereas the numbers in square brackets represent the percent error relative to experiment.

We see in Table 1 that the values of the density and lattice parameters obtained with the AA force field are in good agreement with the experimental ones, with a relative error on the lattice parameters of 1–4% resulting in a 6% relative error for the density. The agreement is worse for the UA force field, which is consistent with the observations reported in the literature.^29^ In particular, the lattice constants for the UA force field are about 4–6% larger than those with the AA force field, resulting in 13% lower density (see Table 1). This decrease in density can be attributed to weaker Coulombic interactions between the mannitol molecules in the UA model, where the magnitudes of the residual charges are on average 10–15% smaller than those in the AA force field, so the attraction between the negatively charged oxygens and the positively charged hydrogens or carbon–hydrogen complexes is weaker compared to those in the AA force field.

From the percent error relative to experiment reported in Table 1, we see that the lattice parameters are consistently underestimated for AA models and overestimated for UA models. From such behavior it follows that the density is larger in AA models and smaller in UA models compared to experiments. This global behavior can indicate a possible optimization strategy for such force fields to recover the correct experimental density, which includes a constant (i.e., the same for every atom type) scaling of the parameters controlling the (nonbonded) interactions, extending the sensitivity analysis presented in de Waard et al.^29^ The comparatively good prediction for the lattice parameters and density we obtained in our work using the AA model may show that such optimization can indeed be performed also for the UA models, instead of considering more refined foce-fields (such as polarizable charges of multipole expansion).

The cleaving simulations were performed in the *NVT* ensemble using the Nose–Hoover thermostat to keep the temperature fixed at 300 K with a temperature coupling time of 100 fs. The initial configurations for these simulations were taken from the final configurations of the above-described anisotropic *NPT* equilibration runs, slightly rescaled to match the average system sizes as reported in Table 1.

The thermodynamic path was obtained using a LAMMPS script, which allows one to run a sequence of calculations and vary the parameters in between. We started by running a calculation with the system in the bulk (λ = 1) configuration. Its last configuration is the starting point of the following run with a modified value of λ. We keep on modifying λ until we reach the value of λ = 0 (i.e., the slab configuration). Each cleaving calculation was performed by dividing the interval [0, 1] into 44 subintervals (for a total of 45 values of λ). The sampling frequency of λ was increased toward the end points of the intervals (i.e., near 0 and 1) and reduced in the middle to allow for a more precise sampling in the end regions. For each value of λ we equilibrated the system for 100 ps and then calculated the work for 500 ps. From this follows that we performed the cleaving of the crystals in 27 ns.

## Results

**Figure 2:**
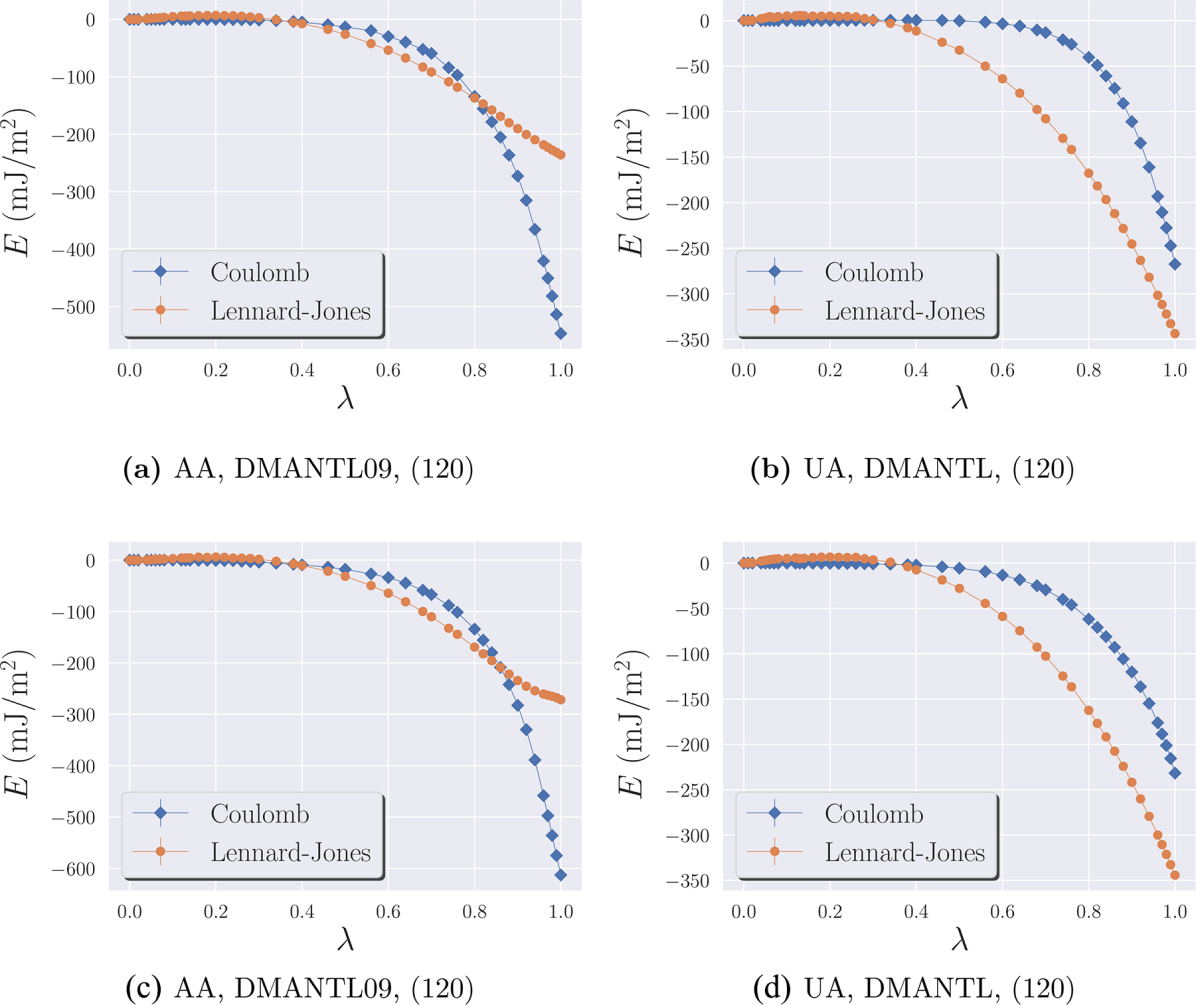
Lennard–Jones (see eq 7a) and Coulombic integrands (see eq 7b) per unit area as functions of λ in the cleaving method for different models and mannitol crystal structures. Surface orientation is (120) (top) and (010) (bottom). Estimated statistical confidence intervals are smaller than the size of the symbols.

First, let us consider the integrands in eqs 7a and 7b, which we label *E*(λ)
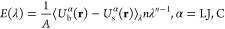
9In Figure 2, we show the dependence of *E* for LJ and Coulomb components on the switching parameter λ for the (120) orientation. The results for other orientations considered in this work are reported in the Supporting Information, SI, (see Figures S.1–S.4).

We notice that the results for the AA and UA models differ by the relative size of the integrands for the Coulomb and LJ components. In the AA model, the magnitude of the Coulomb interactions is much larger than that of the LJ potential, while the opposite is true in the UA model. As mentioned above, this is consistent with the fact that residual charges in the AA model are larger than those in the UA model.

We can also observe, for some of the orientations (e.g., (100) in Figure S.3b), a “bulge” in the LJ integrand at small values of λ, which is caused by the LJ end point catastrophe^8,36^ as discussed in previous sections. Our use of the modified LJ equation in eq 4 and *n* = 4 in eq 3 largely mitigates this effect, so the statistical and integration errors remain under control. This modification works better for the AA model than the UA model, can be explained by the dominating contribution of the Coulombic interactions in the AA model, which, even when scaled by a small λ, do not allow atoms to approach one another close enough for the steep LJ gradient to become a problem.

In Table 2 we report the results for the SFE in all of the cases considered. We see that the two force fields give very different results, with the AA model having a larger SFE compared to the UA model due to the much larger Coulombic contribution.

**Table 2:** Summary of the Results for the Calculation of SFE for Different Force Fields and Orientations of the Mannitol Crystal and Comparison with Experimental Results^a^

UA			
orientation	γ^LJ^	γ^C^	γ
(100)	74.9(1)	37.3(1)	112.2(1)
(010)	74.8(1)	33.7(1)	108.5(1)
(001)	85.3(1)	27.2(1)	112.6(1)
(011)	87.9(1)	29.1(1)	117.0(1)
(120)	78.1(1)	28.5(1)	106.6(1)

AA			
orientation	γ^LJ^	γ^C^	γ
(100)	47.5(1)	137(1)	185(1)
(010)	74.4(2)	82.5(3)	156.9(4)
(001)	9.2(1)	270.6(1)	280.0(1)
(011)	10.7(2)	242.9(2)	253.6(3)
(120)	60.7(1)	76.3(1)	137.0(1)

experimental results^4^			
orientation	γ^*d*^	γ^*p*^	γ
(010)	44.1(6)	12.8(3)	56.9(9)
(011)	39.5(4)	35.4(7)	74.9(1.0)
(120)	43.3(7)	18.6(4)	61.9(1.1)

a All results are in units of mJ/m^2^.

Comparing the simulation results to those from the ICG experiments, we see that the UA force field yields results closer to the experimental values, although still overestimating them by about a factor of 2.

The main contribution to this difference in the AA force field is a very large magnitude of the Coulombic contribution. Indeed, if we draw a parallel between the LJ and the Coulombic contributions in the simulations and the dispersive and polar components in the ICG, respectively, we see that the UA force field yields larger values for both the LJ and the Coulombic contributions compared to the experimental dispersive and polar components, respectively, except for the (011) orientation, where the experimental value of the polar component is larger than the Coulombic contribution from the simulations. In contrast, the Coulomb components for the AA model are almost an order of magnitude larger compared to the experimental value of γ^*p*^.

It is important to highlight here that while a direct comparison of the SFE in calculations and experiments does not require the additional definition of such intermediate quantities as dispersive and polar contributions to the SFE, the identification we considered above between the intermediate quantities requires some care. To what extent the LJ and Coulomb components measured in simulations can be linked with the dispersive and polar components of the IGC measurements needs further investigation on a wide range of molecular crystals. Such investigation will certainly be helpful for improving understanding of these quantities both on the experimental side, by direct linking with computable quantities from molecular models, and on the computational side, to help design better force fields able to correctly describe the surface properties of molecular crystals. This will be the subject of our future work.

From the obtained results, we can also analyze the anisotropy of the SFE, that is, the difference of the SFE values among the different surface orientations. Note that while we measured five orientations in the simulations, the (100) and (001) orientations are not observed experimentally, so they are expected to have higher SFEs compared to other orientations. In the simulation of with both UA and AA force fields, we see that, consistent with experiments, the SFEs of the (010) and (120) surfaces are smaller than those of the (100) and (001) surfaces, with (120) having the smallest SFE. However, the (011) surfaces has a rather large SFE, especially with the AA force field, which is caused by a very large Coulombic contribution. This overall mixed qualitative agreement between simulations and experiments brings us to the conclusion that more research is needed to develop force fields for organic molecular crystals that would be better at predicting the surface properties of such materials.

## Conclusions

In this work, we extended the cleaving method to calculate the surface free energy of molecular crystals and applied it to β-d-mannitol by two different force fields. Three different structures of the mannitol crystal available in the Cambridge Structural Database (DMANTL, DMANTL07, and DMANTL09) were used in the initial construction of the mannitol crystal, but only one of these structures (DMANTL for the united-atoms (UA) and DMANTL09 for the all-atoms (AA) force field) was found to be defect free after equilibration at 300 K and zero pressure. For each force field, the SFE was calculated for five different orientations of the crystal surface. The computations were implemented in LAMMPS.

From the obtained results, we observe a substantial difference in the computed SFE between the all-atoms (AA) and the united-atoms (UA) force fields. Even though the AA force field predicts a crystal structure and density closer to the experimental values, it significantly overestimates the SFE compared to the experimental results from the IGC measurements. The reason for such an overestimation is in the relatively large residual charges and thus much stronger Coulombic attraction between mannitol molecules described by the AA force field. This attraction appears to be needed to obtain lattice parameters closer to the experimental values, yet it results in a significant overestimation of the SFE by this force field.

In contrast, the UA force field, which would normally be expected to yield worse predictions due to a coarser model and a correspondingly smaller set of model parameters, predicts the SFE in better agreement with the experimental values. In addition, if the IGC-measured dispersive and polar components of the SFE can be associated, respectively, with the LJ and Coulombic interactions in the model force field, there is a reasonable agreement between the simulations and the experiments in the case of the UA force field, while the AA force field yields a very large overestimation (by about and order of magnitude) of the polar component.

On the basis of the presented results, we conclude that while some qualitative agreement between the results of the UA force field and the IGC experiments can be observed, more work is necessary to investigate the effects of the force field topology and parameters on the surface properties of molecular crystals. In particular, optimization of the effective interactions described by the classical force fields (which include the Lennard–Jones and Coulomb parameters) would be required to obtain an improved description of the surface properties of such systems alongside their bulk properties. Development of force fields which better capture such diverse properties of materials is very challenging, but it is of significant technological importance in many industrial and manufacturing applications.

## Supporting Information Available

Additional details of the setup of the MD calculations and the cleaving model; results of the work along the cleaving path for the crystallographic directions not included in the main paper (PDF)
