# Supplementary material for: Cleaving Method for Molecular Crystals and Its Application to Calculation of the Surface Free Energy of Crystalline β-d-Mannitol at Room Temperature
Source: J Phys Chem A. 2022 Mar 24;126(13):2134–41. doi: 10.1021/acs.jpca.2c00604 (PMC9007450; doi:10.1021/acs.jpca.2c00604)
Supplement: Supplementary file 1 — jp2c00604_si_001.pdf [file jp2c00604_si_001.pdf]

Supporting Information for "Cleaving Method for Molecular Crystals and its Application to Calculation of Surface Free Energy of Crystalline β-D-Mannitol at Room Temperature"

Nicodemo Di Pasquale*,†,‡ and Ruslan L. Davidchack†

†*School of Computing and Mathematical Sciences, University of Leicester, University Rd, Leicester LE1 7RH, UK*

‡*Present address: Department of Chemical Engineering and Analytical Science, University of Manchester, Manchester M13 9AL, United Kingdom*

E-mail: nicodemo.dipasquale@manchester.ac.uk

Equilibration of the system

In the simulation, no constraints were used for the molecules (i.e., all the bonds are flexible). The weighting coefficient for the bonds, angles and dihedrals, were all set in the simulations using the LAMMPS keyword:

```
special_bonds lj 0.0 0.0 0.5 coul 0.0 0.0 1.0
```

which sets zero weight for LJ and Coulombic interactions for atoms connected by bonds (1-2) and angles (1-3) and a weight equal to 0.5 for LJ and 1 for Coulomb for atoms connected by 1-4 interactions.

I order to check the stability of the configuration against the chosen force-fields, and that the simulation setup does not cause deformation of the crystal structure not observed in experiments, we performed a series of calculation in the NσT ensemble at a pressure of 1 bar and a temperature of 300 K, with non-isotropic coupling, i.e., the six components of the pressure tensor were allowed to vary independently. We used a pressure coupling time of 1 ps and a temperature coupling time of 100 fs, and let the simulations run for a total of 10 ns. For this calculation, the box type was changed in LAMMPS from an orthogonal to a triclinic. The biggest deviation from orthogonality obtained for the angles between the axes of the unit cell was about 2° for both AA and UA model, This demonstrated that the simulated structures were indeed orthorhombic. In subsequent simulations, the systems were equilibrated using anisotropic barostat with diagonal pressure tensor, in which the diagonal components are independently equilibrated .

During the equilibration of different initial structures in both AA and UA force fields, we carefully monitored the deviation of the crystal structures from their average configurations. Some structures developed defects during the equilibration and had to be excluded from the subsequent cleaving calculations for the determination of the surface free energy (SFE). Only the structures that remained stable in their crystalline configuration over the whole

simulation volume were retained.

Cleaving calculations

In the configuration we are considering, the cleaving plane is not within the box but on its ends, that is to say the scaled interactions are calculated *only* between the molecules inside the box and their periodic images outside the box. In order to achieve this, we divide the molecules within the box in two types, based on their position with respect the center of the box. Due to their finite size, the molecules can cross the plane positioned in the center of the box and, to give a unique label to each of them, the assignment was done using their Center of Mass with respect the position in the box.

In this way, we know that if a molecule is interacting with a molecule on the same side of the cleaving plane (thus using the full interactions not scaled by λ) or with molecules on the other side of the cleaving plane, for which we need to consider scaled interactions. In fig. S.1, we give a sketch of the arrangement of the molecules leading to a scaled and non-scaled interactions. This setup is needed since molecules have finite size and they can cross the cleaving plane. See for example the molecule c' in fig. S.1 which is the image of molecule c of Type 1. In this case, it needs to interact with scaled interaction (through λ) with neighbouring molecules not on its side of the plane (such as a and b) even though c' is partially on “the same side” of the cleaving plane of molecules a and b. The fact that c' is of Type 1, while both a and b are of Type 2, allows the algorithm to assign the correct scaling for their interactions.

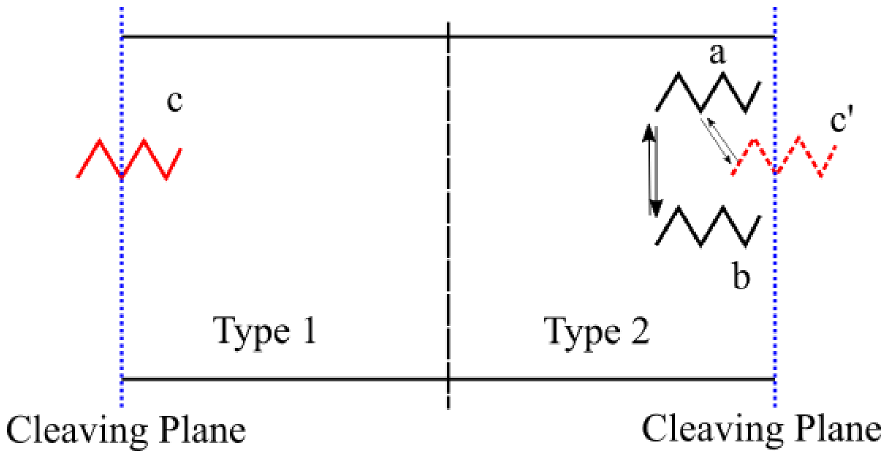

Figure S.1: Sketch of the calculation of the scaled and non-scaled interactions. Molecules a and b are of Type 2 and interact with the full interactions. The molecule c' is a periodic image of the molecule c, which is of Type 1. Its interactions with molecules a and b are scaled by λ.

Additional Results for cleaving

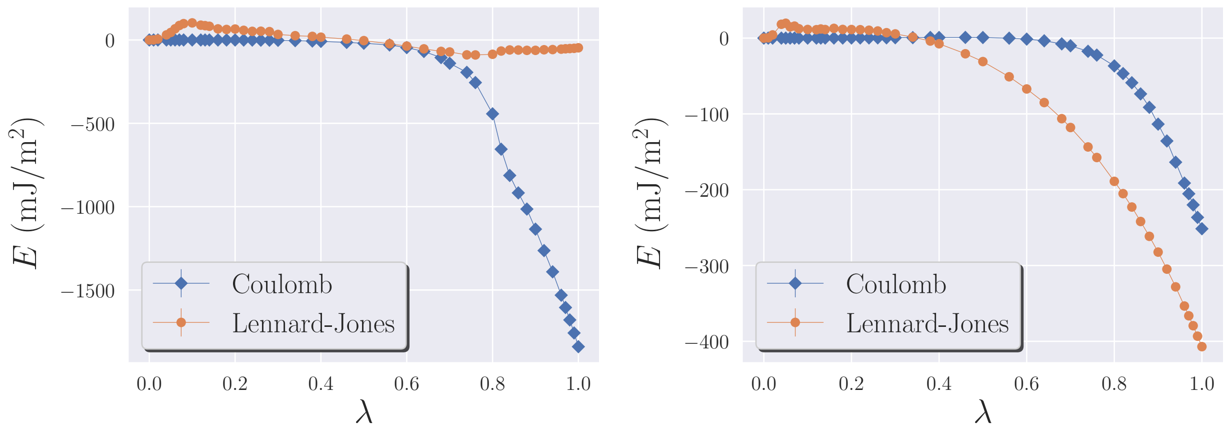

(a) AA, DMANTL09, (001)

(b) UA, DMANTL, (001)

Figure S.2: Lennard-Jones and Coulombic integrands in Eq. (7) (in the main paper) per unit area as functions of λ in the cleaving method for different models and mannitol crystal structures. The estimated statistical confidence intervals are smaller than the size of the symbols.

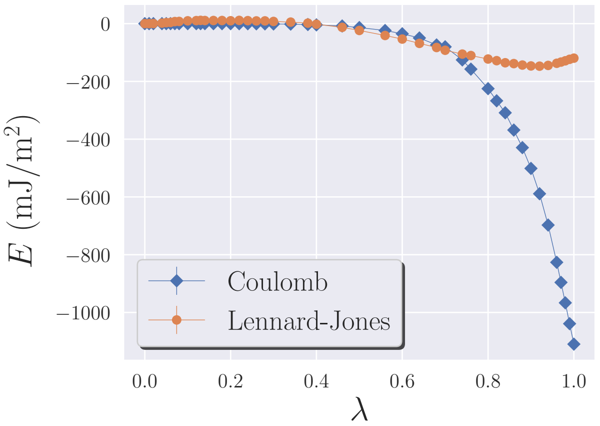

(a) AA, DMANTL09, (100)

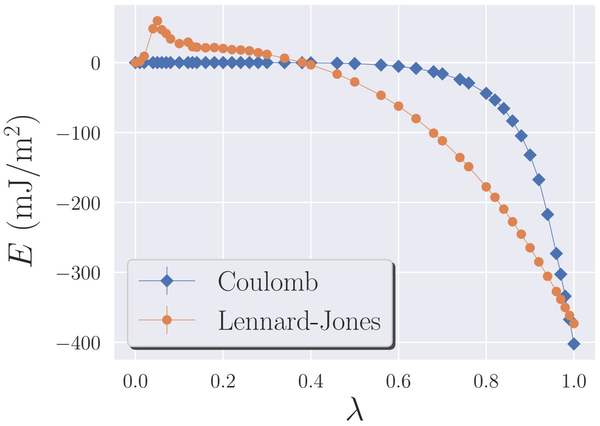

(b) UA, DMANTL, (100)

Figure S.3: Lennard-Jones and Coulombic integrands in Eq. (7) (in the main paper) per unit area as functions of λ in the cleaving method for different models and mannitol crystal structures. The estimated statistical confidence intervals are smaller than the size of the symbols.

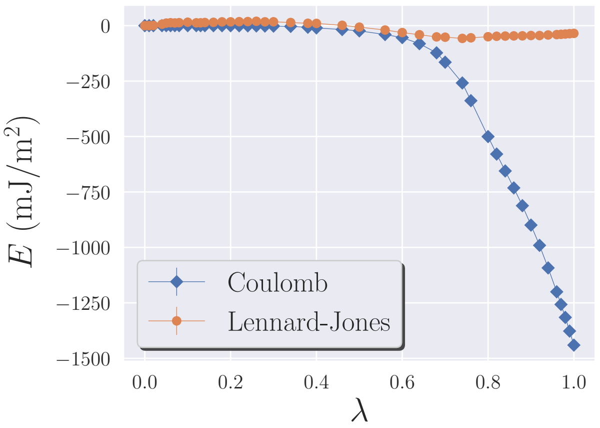

(a) AA, DMANTL09, (011)

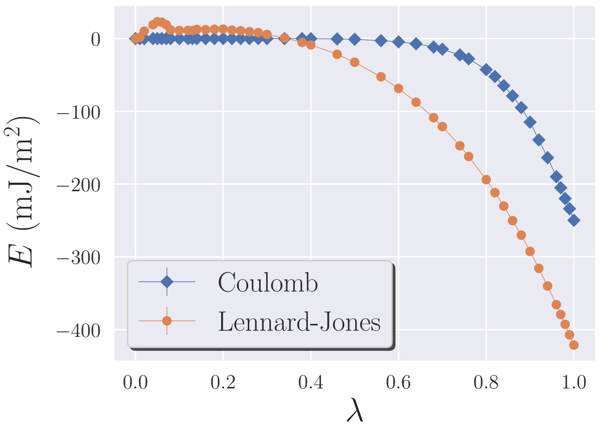

(b) UA, DMANTL, (011)

Figure S.4: Lennard-Jones and Coulombic integrands in Eq. (7) (in the main paper) per unit area as functions of λ in the cleaving method for different models and mannitol crystal structures. The estimated statistical confidence intervals are smaller than the size of the symbols.
